# “Something very taboo”: a qualitative exploration of beliefs, barriers, and recommendations for improving mental health care and access for Hispanic adults in the Paso del Norte U.S.-Mexico border region

**DOI:** 10.3389/fpubh.2023.1134076

**Published:** 2023-06-01

**Authors:** Jason Mallonee, Rosa Escalante, Eden Hernandez Robles, Christal Tucker

**Affiliations:** ^1^Department of Social Work, College of Health Sciences, The University of Texas at El Paso, El Paso, TX, United States; ^2^Worden School of Social Service, Our Lady of the Lake University, San Antonio, TX, United States; ^3^The Kelly Center for Hunger Relief, El Paso, TX, United States

**Keywords:** Hispanic, Latino, mental health, help-seeking, barriers, stigma, U.S.-Mexico border region

## Abstract

**Background:**

Hispanic adults with mental health conditions in the United States experience disproportionate access to and utilization of professional mental health treatment. This is believed to be in part due to systemic barriers and challenges, difficulty accessing care, cultural factors, and stigma. Studies to date have failed to examine these specific factors within the unique context of the Paso del Norte U.S.-Mexico border region.

**Methods:**

For this study, 25 Hispanic adults identifying primarily of Mexican descent participated in four focus groups exploring these topics. Three groups were facilitated in Spanish and one group in both English and Spanish. Focus groups followed a semi-structured format eliciting perspectives on mental health and mental illness, help-seeking, barriers and facilitators of help-seeking and treatment access, and recommendations for mental health agencies and providers.

**Results:**

Qualitative data analysis yielded the following themes: understanding of mental health and help-seeking; barriers to accessing care; mental health treatment facilitators; and recommendations for agencies, providers, and researchers.

**Conclusion:**

Findings from this study support the need for innovative mental health engagement strategies to reduce stigma, increase understanding of mental health, foster support systems, reduce individual and systemic barriers to seeking and accessing care, and to continue to engage communities in mental health outreach and research.

## 1. Introduction

In 2020, only 35.1% of Hispanic^1^ adults with mental health conditions in the United States received professional treatment, compared to 51.8% of non-Hispanic white adults (1). Less frequent treatment utilization, shorter episodes of care, inadequate care, and fewer referrals to psychotherapy characterize Hispanic adults’ experience with mental health services (2). Less frequent service utilization is associated with a range of deleterious outcomes, including poorer physical health (3), co-occurring substance use (4), higher rates of unemployment (1), homelessness (5), and incarceration (6). Given the disproportionate prevalence of unresolved mental health conditions for Hispanic adults in the United States and the associated adverse outcomes, it is critical that researchers study pathways to care that potentially reduce barriers and increase access to services.

A growing body of literature provides broad insights into mental health treatment barriers. Poverty and lack of insurance (7), linguistic and transportation barriers (8), and disproportional access to care (9) are some well documented systemic barriers. Stigma-related factors like cultural beliefs around mental illness and help-seeking further reduce the likelihood of Hispanic adults seeking and engaging in professional mental health treatment (10–12). For Hispanic adults, lower socioeconomic status, and an inability to meet basic needs are associated with less frequent patterns of help-seeking and lower rates of mental health treatment utilization (13, 14). Food insecurity is associated with poorer mental health outcomes, including increased rates of depression and stress (15), impaired mental health status (16), and increased mental health diagnoses (17).

One shortcoming of existing research on help-seeking and treatment engagement for Hispanic adults is that studies frequently collapse groups from various backgrounds and nationalities together, or exclude Spanish-speaking clients altogether (13). In order for intervention efforts to more effectively reduce disparities for Hispanic populations, research is needed that illuminates the unique experiences and perspectives of subgroups within Hispanic communities. While valuable research is emerging on mental health care for Hispanic adults of Mexican descent on the U.S.-Mexico border (18, 19), the border itself reflects a vast range of diversity along its 1,954 miles. As such, interventions that are tailored to regional perspectives and needs may be more effective at reducing mental health disparities at the community level. This study aims to fill the identified gaps by eliciting the perspectives of Hispanic adults primarily of Mexican descent located in the Paso del Norte region of the U.S.-Mexico border through community-engaged, culturally responsive, and linguistically appropriate qualitative research.

Recognizing the association between the inability to meet basic needs and less frequent mental health help-seeking, as well as the relationship between food insecurity and poorer mental health outcomes, this study recruited participants from one of the largest food pantries in the Paso del Norte region. The specific aims of this study were to: (1) better understand regional perspectives on mental health and mental illness, (2) identify regional and cultural barriers to mental health help-seeking and treatment engagement from the perspective of community members who may be experiencing unresolved mental health conditions, and (3) to elicit community member recommendations for mental health agencies and providers. This study is situated within a larger research program aimed at reducing mental health disparities for Hispanic adults living in the Paso del Norte U.S.-Mexico border region. Findings from this study will inform the development of a community-grounded mental health engagement program, which will next be pilot tested for acceptability, feasibility, and preliminary efficacy.

## 2. Materials and methods

For this qualitative exploratory study, data were collected from four focus groups conducted during April 2022 with 25 participants. Researchers aimed for focus groups consisting of five to eight participants each (20), although remained flexible. The focus groups consisted consecutively of nine participants, eight participants, five participants, and three participants. The first three focus groups were conducted entirely in Spanish, and the final focus group was conducted in both Spanish and English simultaneously. The primary moderator for this group was able to present questions in both English and Spanish and translate participant contributions in real time given the small size of the focus group. All screening interviews and focus groups were conducted by trained graduate research assistants under the guidance of the lead investigator, a doctoral prepared licensed clinical social worker with expertise in clinical interviewing and interventions.

Participants were recruited through convenience sampling at one of the region’s largest food pantries. Agency staff assisted in recruitment efforts by distributing a paper flyer in both English and Spanish with the study information to individuals receiving services at the food pantry. To be eligible to participate in this study, individuals had to be 18 years or older, identify as Hispanic, and live in El Paso County, Texas. Those who were interested in participating were tracked using a participant interest log that was provided to the research team. Research team members and agency staff contacted interested participants by phone or in person to schedule the screening interviews and focus groups. Focus groups for participants who met selection criteria and provided informed consent were conducted directly after their screening interviews. All individuals who expressed an interest in participating in the focus groups met the inclusion criteria detailed above; therefore, no individuals were excluded based on not meeting study eligibility criteria.

For the first focus group, three scheduled participants arrived at the scheduled time, with one bringing an additional person interested in participating. As this focus group took place during busy hours at the agency, five additional participants expressed interest that day, and after screening all of these potential participants for eligibility, all nine were included in the focus group. For the second group, eight of the nine scheduled participants arrived at the scheduled time, screened eligible to participate, and were included in the focus group. For the third focus group, five of the seven scheduled participants arrived at the scheduled time, screened eligible to participate, and were included in the focus group. For the fourth focus group, four of the eight scheduled participants arrived at the scheduled time and screened eligible to participate. One of these participants decided not to participate, so three were included in this focus group. Research team members reached out to all who were not present at the scheduled time of their screening interview and focus group to attempt to reschedule, but all were either no longer interested or not reachable by phone.

Once a participant was screened eligible to participate and provided informed consent, sociodemographic data and information related to mental health treatment history were collected through a self-administered paper survey. Upon all focus group members’ completion of this survey, focus groups were facilitated by a primary moderator while a secondary moderator took notes. All focus groups were recorded using two audio recording devices in case one device malfunctioned. Focus groups were conducted using a semi-structured focus group guide developed by the research team in collaboration with agency staff and in consultation with cultural content experts. Question domains included (1) perceptions of mental health and mental illness, (2) help-seeking behaviors, (3) barriers and facilitators of help-seeking and treatment access, and (4) recommendations for mental health agencies and providers. Questions were structured in the focus group guide to move from general to specific (20), with optional follow-up prompts the moderator could use to help guide the conversation. Snacks, beverages, and a notepad with pen were provided to all focus group participants. Focus groups began with introductions and establishment of community agreements prior to engaging in dialogue around study subject matter. Focus groups ranged from 62 to 89 min in duration.

The Institutional Review Board at The University of Texas at El Paso approved the research protocol. All participants provided their written informed consent to participate in this study.

### 2.1. Data analysis

Descriptive statistics were utilized to present study sample characteristics and previous experience with mental health help-seeking and treatment utilization. Qualitative data analysis reflected Marshall and Rossman’s (21) seven-step process, including (1) organization of the data, (2) immersion in the data, (3) generation of themes and subthemes, (4) coding of the data, (5) interpretation of data through analytic memos, (6) consideration of alternate understandings, and (7) dissemination of study findings. Data from focus groups were transcribed and translated into English prior to analysis. Focus group data were then placed into a Word document with minor revisions to correct spelling and other typos. Transcripts were organized both by focus group and by the following question clusters: (1) perceptions of mental health and mental illness, (2) help-seeking behaviors, (3) barriers and facilitators of help-seeking and treatment access, and (4) recommendations for mental health agencies and providers. Although organizing the data conceptually by question cluster can be viewed as a form of deductive coding (22), researchers were encouraged to explore and examine the raw data more naturalistically utilizing an inductive process to determine actual themes and subthemes. Given some overlap between question cluster organization and overarching themes in this study’s findings, the data analysis process reflects more of a mixed deductive-inductive approach, which is common in thematic analysis (22). A secondary content analysis was conducted to determine the number of participants speaking to each theme and subtheme (23).

In order to identify salient themes and subthemes, two researchers independently read and reread the transcripts as organized by focus group. They then read and reread the transcripts organized by question cluster, using open coding to identify patterns and themes throughout the transcripts (24). This resulted in each researcher independently developing a set of themes and subthemes. These two researchers negotiated a coding framework utilizing Padgett’s (23) process of “consensual validation” (p. 250), an iterative and flexible process where researchers present, compare, and collaboratively revise the coding framework to reach consensus, all while grounding the process in the data. One researcher coded the transcripts using this agreed upon coding framework. To triangulate the data and minimize researcher bias, two additional researchers – one an expert in the content area and the other a community provider – reviewed the raw data and coding and suggested additional considerations and revisions. All four researchers participated in the writing and interpretation of findings. All analysis was conducted in Microsoft Word and Excel. As part of participant checking, findings were reviewed with agency leadership where participants were recruited from. Feedback was provided throughout the analysis, and a member of agency leadership collaborated on study design, data analysis, and drafting of this manuscript.

### 2.2. Participant characteristics

All participants identified as Hispanic and were provided with an option to elaborate on their ethnicity and heritage. Most participants indicated they were of Mexican descent (68%), with two participants (8%) indicating a mixed ethnicity – one as Mexican American/American Indian and the second as Mexican/Irish. One participant (4%) identified as “Chicano.” Seven participants (28%) chose not to expand on their ethnicity and heritage. Roughly two-thirds (64%) of participants identified as female, with the remaining identifying as male (32%) or preferred not to answer (4%). Participants ranged from 32 to 75 years of age, with a mean age of 40.04 (SD = 11.17 years). Forty percent were married, and the remainder were either single (28%), separated or divorced (20%), widowed (4%), or declined to answer (8%). Participants reflected a range of education levels, with the last schooling completed as elementary school (12%), middle school (12%), high school (32%), general educational development (GED) (16%), some college (4%), a bachelor’s degree (4%), and a master’s degree (8%). Three participants (12%) declined to provide information on their educational background. Household annual income ranged from $0 (20%), $1–$9,999 (20%), $10,000–$24,999 (20%), $25,000–$49,999 (8%), and $50,000–$74,999 (8%). Six participants (24%) declined to provide information related to family income. Roughly half (48%) reported needing mental/behavioral health services in the past; 10 participants (40%) reported utilizing services, including counseling for mental health, depression, and anxiety, while two (8%) reported using psychiatric medication services. Seven of the 10 participants who had previously utilized services were satisfied with the quality of services they received.

## 3. Results

**Figure 1:**
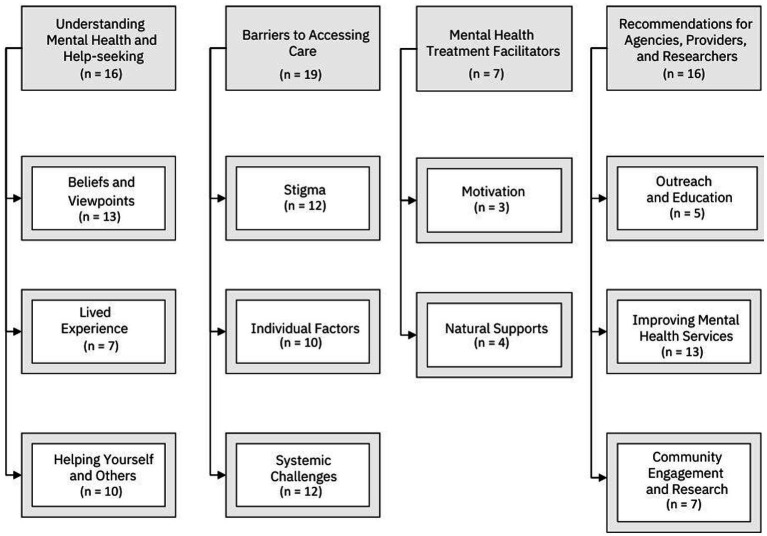
Themes and subthemes.

**Figure 2:**
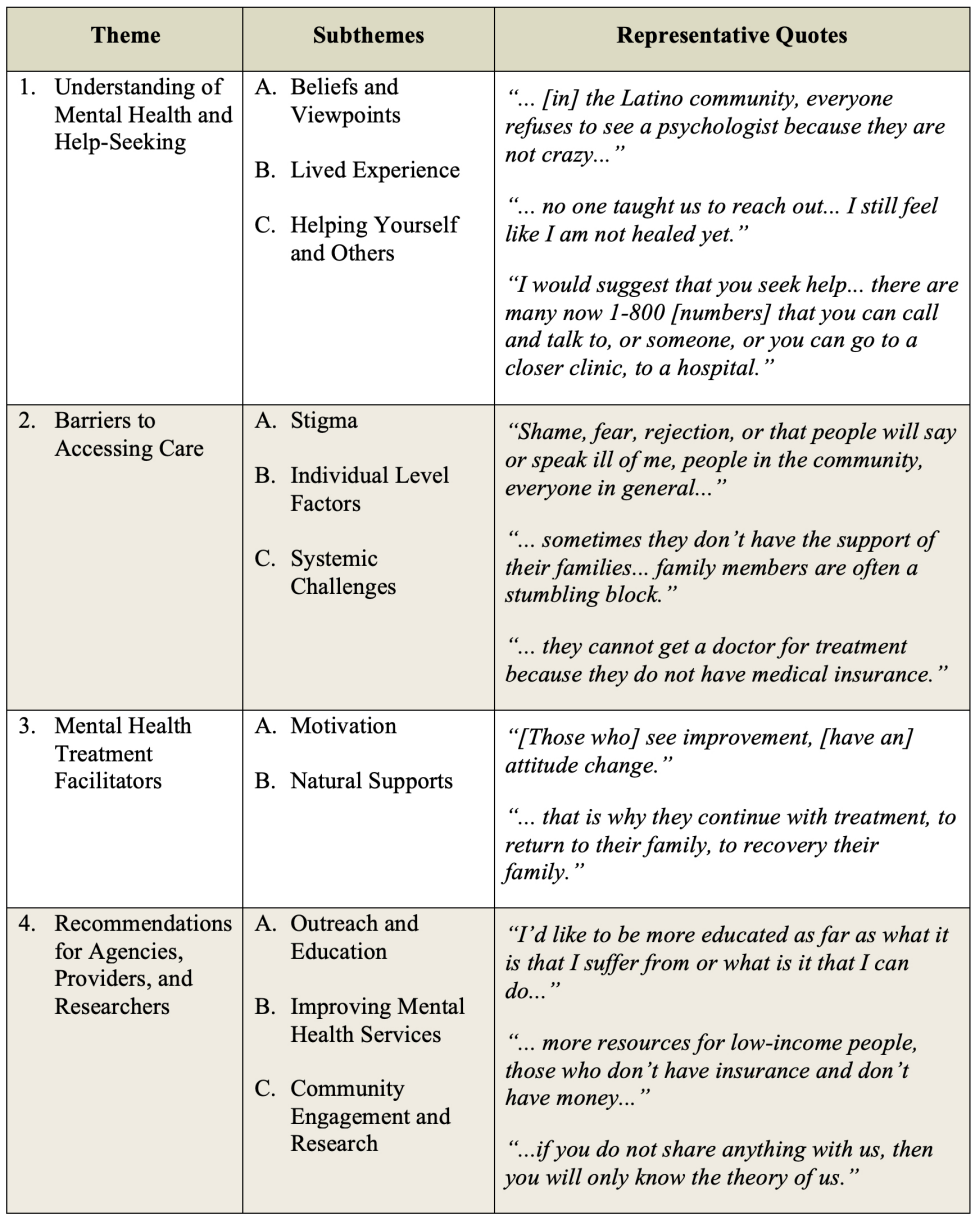
Representative quotes.

The specific aims of this study were to: (1) better understand regional perspectives on mental health and mental illness, (2) identify regional and cultural barriers to mental health help-seeking and treatment engagement from the perspective of community members who may be experiencing unresolved mental health conditions, and (3) to elicit community member recommendations for mental health agencies and providers. Qualitative data analysis yielded the following themes and subthemes as they relate to these aims: (1) understanding of mental health and help-seeking (*n* = 16) with subthemes of beliefs and viewpoints (*n* = 13), lived experience (*n* = 7), and helping yourself and others (*n* = 10) (aim 1); (2) barriers to accessing care (*n* = 19) with subthemes of stigma (*n* = 12), individual factors (*n* = 10), and systemic challenges (*n* = 12) (aim 2); (3) mental health treatment facilitators (*n* = 7) with subthemes of motivation (*n* = 3) and natural supports (*n* = 4) (aim 2); and (4) recommendations for agencies, providers, and researchers (*n* = 16) with subthemes of outreach and education (*n* = 5), improving mental health services (*n* = 13), and community engagement and research (*n* = 7) (aim 3). Please see Figure 1 below for a visualization of these themes and subthemes. Figure 2 provides some representative quotes for each theme and accompanying subthemes.

### 3.1. Theme: understanding of mental health and help-seeking (*n* = 16)

Participant responses reflected a broad range of views on mental health and help-seeking, with cultural attitudes, values, and stigma around mental health influencing perceptions of mental health and treatment as a common thread across groups. Many participants shared strategies for dealing with mental health conditions rooted in their own experiences and observations. Others shared their evolution in thinking around mental health and their distrust of mental health service delivery systems. These perspectives are reflected in the following subthemes.

#### 3.1.1. Subtheme: beliefs and viewpoints (*n* = 13)

Cultural beliefs were prominent throughout the focus group discussions. One participant spoke directly of gender role expectations in Mexican culture, describing needing mental health treatment as “something very taboo, something that is not needed if you are a man. You are a man and you do not need help so they can control your mind.” This participant elaborated that as men age, they begin to see mental health as “something that is required because we are not always strong.” Others spoke more broadly about cultural stigma, with one participant sharing:

My experience was that I was in Mexico and never heard the word psychologist and then I come to the United States and… this one goes to the psychologist, and what is that and why do they go then, because they are crazy and I’m not crazy… [in] the Latino community, everyone refuses to see a psychologist because they are not crazy… I have to admit I went once and I’m embarrassed to tell my friends for the same reason the community is closed to that.

Stigma, fear of being negatively perceived by others, and judgment permeated participants’ conceptualization of mental health, mental illness, and substance use. One participant shared, “the culture of Mexico and the one here is very different… I’m telling you about values… if they teach you good values, you are not going to grasp that [drugs].” Another participant shared, “When they hear mental illness, they already think they have a bad brain. So mental health is crazy. Already because one has nerves it is already mental. No… no… no… that’s why I do not say anything to doctors.” One participant described mental illness as “something is not right inside you,” while another observed:

Back then it was mental retardation, so that is politically incorrect now… the whole mental health was, you know, you are a retard. In my family it was really a no-no, you really didn’t suffer from that kind of stuff. If you did, go figure it out… Nowadays, people are starting to understand that it’s a broad range of things… there’s a lot of things we suffer from.

Some participants viewed mental health conditions as something they have observed happen to other people. In reflecting on knowing people with depression, Alzheimer’s, and schizophrenia, one participant stated, “what comes to mind is someone who needs help.” Another participant shared, “these people need a lot of help… because many times we judge people, this person is sick in his mind… they are not bad because they are people who are in a bad mind.” This participant continued, “we should not be afraid because we should help our neighbor because the biggest thing can be for that person and tomorrow it can be us.”

Several participants compared mental health conditions with physical health. One participant shared, “When you are very depressed, they say that if you do not talk to someone or a psychologist to help you understand what is happening, that’s when illnesses come out, you start to get sick, with this or that.” Another participant shared, “Depression is like cancer that eats away little by little.” This participant stressed the importance of treating mental health problems, “if you have mental problems, do not be embarrassed, it’s a disease that you should seek treatment for.” Another participant similarly shared the importance of seeking help early, “before the problem gets longer or complicated… I think there are consequences if they do not go, well, they will struggle more.” One participant shared:

I think of [mental health treatment] as a great wasted tool… you have a fever, you go to the doctor, and you go to a normal doctor, but if you have a [mental health] problem, there are doctors who can help you or specialized people like psychologists or psychiatrists… most of the people I’ve dealt with do think that it’s useless and I came to think that too until I found out that it’s not.

#### 3.1.2. Subtheme: lived experience (*n* = 7)

In addition to a broader understanding of mental health and mental illness, several participants shared their personal experiences with mental health. One participant shared:

Depression is very ugly, only those who have been through it know this and sometimes one doesn’t talk about it either. High blood pressure, multiple illnesses, it brought asthma to me… You feel you are inside a glass and here you stay there and there is no way to let go, there is no way to say that you feel bad… nothing matters… does not matter your life, you do not care about the lives of others.

Another participant validated the potential severity of mental health challenges, “It makes me very sad to know when someone commits suicide and… there was no one to help him, because that’s what depression will lead to, suicide… he/she did not find someone quickly.”

Some participants shared the impact of their own traumatic experiences, with one sharing, “I suffer from past experiences, whether it be doping up or getting beat up by my parents. It’s the trauma that I went through.” This participant shared frustration with professional treatment responses, “It is what it is nowadays, they can give you a tic tac for anything, that is all they want to do is keep you on some meds.” He continued, “I myself for a long time did not want to admit that I suffered from mental health because obviously the stigma behind it, but now it is a little more accepted… I do take part in my therapy.” This participant also shared about his recovery journey, “I’ve been suffering from mental health for obviously quite some time… I started self-medicating, so I have been in active addiction… for like 30 some odd years. I am in recovery now.”

Other participants shared fears about talking about their experience with mental health. One stated, “I did not tell my friends because they thought I was crazy.” Another shared, “Before people would not talk about it, your dad drinks, okay shut up, do not say nothing, leave it, go to sleep, tomorrow is another day.” This participant continued:

That is where the abuse is at, and the whole family just want to wait, don’t say nothing… no one taught us to reach out… I still feel like I am not healed yet. I do not think I will ever be healed. I feel like with my past trauma, and my trauma bag is so full, that sometimes I just break down, it’s too much.

Another participant shared, “My daughter has also seen me very bad when we have video calls, sometimes I am so very sad and I cry.” This participant shared her own challenges with accessing services, “… my daughter tells me she’s going to Juárez, mom, if they cannot get help there, go to Juárez, look for a psychologist… but I’m afraid to go to Juárez.”

For some, the conversation about mental health elicited experiences observed in friends and family. One participant described, “I also know another person who was in the army when he was very young and everything that happened to him… he has problems because he remembers everything he lived through.” Another shared, “I learned a lot with my daughter… she went to therapy and recovered a lot, to the psychiatrist and she is very well… but she was not out of her mind.” These personal and shared experiences reflected a shift in thinking about mental health for focus group participants, which was also reflected in their discussion on how they might help themselves or someone else who may be suffering from a mental health condition.

#### 3.1.3. Subtheme: helping yourself and others (*n* = 10)

Several participants shared strategies for helping others address their mental health. One shared, “I would try to sit down and talk with him and listen to him. What is his problem? What does he feel? … and maybe with that talk, he can release a little bit of the pressure he feels.” Another participant responded:

It is about helping to provide them with a tool, to give them a little punch so that they go to help their family member or themselves if they are going through that situation… that they do not let these things pass lightly because over time everything gets worse. I would suggest that you seek help, because it is very sad to see them lock themselves in their world of… not asking a friend for advice… or seeking help for the simple fact of fear that they will be judged.

One participant described a nuanced approach to helping others:

I have helped people by guiding them to therapy, but what I have seen is that you cannot arrive and say you don’t know what you need is therapy, listening to see how his point of view is going and at some point, looking for a way to guide him.

Another participant shared, “I would suggest you seek help… also help you find help, a center or find a place where you can,” continuing, “there are many now 1–800 [numbers] that you can call and talk to someone or you can go to a closer clinic, to a hospital.”

Several participants described the importance of having support of family, friends, and a spiritual connection. One shared, “I think that first of all, one should put God in the first place and then… counseling that person, talk to them, give him love, give him support.” He continued, “… you have to have a good friend or a good person… with whom to talk.” Another shared, “There are many people who call mental problems that the only thing they need is affection, attention from relatives.” One participant shared the challenges with trying to help others:

I realize that you can’t make someone do something you do not want to do… I suggest it to my friends but I can’t force them to, I want them to but I can’t force them to do something… That is the choice they have to make.

One participant shared the power of being a living example, “… with my own family and relatives… whoever knows my story, I tell them, if I can do it, you can do it, I am living proof right here, okay I am not perfect, but I did it.” This sense of a collectivist orientation toward helping yourself and others was clearly reflected in the personal stories that participants shared.

### 3.2. Theme: barriers to accessing care (*n* = 19)

Although some of the barriers to accessing care experienced by focus group participants were alluded to when describing their understandings of mental health and help-seeking, including stigma and difficulty accessing care, these were elaborated upon during directed dialogue around barriers to help-seeking and accessing care.

#### 3.2.1. Subtheme: stigma (*n* = 12)

Stigma, shame, and fear of how they might be perceived were prominent themes in discussions of barriers to accessing care. In one focus group, multiple participants spoke collectively about this barrier, “Shame, fear, rejection or that people will say or speak ill of me, people in the community, everyone in general.” This reflection came up at the individual level in all focus groups. One participant shared, “…the other [barrier] is shame… they feel like they are going to be told they are crazy.” Another added, “We ourselves judge what we hear in the community… she may be mentally ill, we do not know… when you say she is crazy… that is why they are afraid to… ask for help.” This participant later clarified, “It is not that fear is so much, his brain at that moment is blocked, that is, he is not thinking, that is why we need the help of the other friends and relatives.” One participant shared, “They say the man is crazy. He went to ask for help, and there are about 27 places where they can help him, but it is the pride and shame that one does not want.” Another participant shared:

We know where they can help you and we keep quiet, for the simple fact that no, they will say that I am nosy or maybe they will judge me badly… it is the fear of being judged or fear of the family itself that is the first to judge, without knowing the reasons why… because the family is the first to judge.

Another described this as “the fear, the rejection of the family that he is crazy, mentally ill and oh, we have to separate from him.” One participant shared a similar sentiment:

The ego is also a very strong barrier… because of the way we are, how we developed in Mexico, that way, yes, what will they say and what is the neighbor going to say, what is he going to say? … what do you think, that her son went to a psychologist or I knew that so and so’s girlfriend brings problems and there is a lot of talk in society.

A different participant concurred:

I think the number one [barrier] would be bullying. He’s going to that; it means he’s crazy and the community is already starting to make fun of him. To the person who needs help and goes to a mental health center, the other people later describe him as crazy and that is why he is going there, and they bully him.

Although shame and stigma were prominent themes in the discussion of barriers, one participant offered a glimmer of hope in how people think of those with mental health conditions:

There was something obviously wrong, you are not all there, you are not playing with the full deck, right, so that is the stigma. Nowadays, obviously people become more educated about and know that… you suffer from a lot of illnesses, anxiety being one of the main ones.

#### 3.2.2. Subtheme: individual factors (*n* = 10)

Participants identified various individual factors that shape help-seeking or the ability to access mental health services. Some of these were related to the individual’s circumstances. One participant identified poverty as “the number one reason” for their inability to access services. Another added, “[a] lack of time, single mothers cannot go because they have to work. That is also lack of time… work schedules… well-being, living in poverty.” Another similarly shared:

They make the excuse of time. It’s just that I don’t have time, it’s that right now I have another more important appointment and I’ll attend to that later. Time is its worst enemy… it is the most common excuse, that there is no time.

Other participants shared about low motivation, acceptance of having a mental health condition, and lack of progress as barriers. One shared, “… if you do not continue the treatment, it’s because it’s not helping you.” Another stated:

He went two or three times due to pressure from his family, due to pressure from his wife and he went, but he never had the intention of… never accepted that he had a problem. The internal reasons such as fear, especially fear of facing their own responsibility.

One participant identified “lack of support” as a barrier, while another shared, “sometimes they do not have the support of their families… family members are often a stumbling block.”

A lack of awareness of mental health conditions was also understood as a significant barrier for many participants. One described this as “the lack of education… if we were well educated like the American culture, for example, that it is very normal that you pick up the child from school and take him to the psychologist.” One participant described:

What happens is there is a lack of information… people need to have help with a little information and a lot of it confuses their mind, that is why they do not seek help… there are many people who need help, and more so with this that COVID has just passed. A lot of stress, confusion, many attacks of hysteria, and it was all for the same reason that there is no information and that is why many people shut down and do not want to receive help, even if they need it.

Another participant shared:

If he were educated by now, it would be another decision that it is for his own good and that they can help him… the person does not go because of fear, because [he] is not informed… the information is not made known to many people who do not have access to information like this, but the help does exist.

Some participants also identified their fear of treatments and providers as barriers. One described, “they do not feel good with the medicine… instead of helping, [they] make them feel worse. They do not want to follow treatment.” Lastly, a participant described:

Even if I felt half crazy, I still don’t go to a psychiatrist. I don’t go to a psychiatrist because I’m afraid of them… I just tell them that I was sad, that if I wanted to die, they already wanted to send me to the asylum, well forget it, no… I no longer tell them.

#### 3.2.3. Subtheme: systemic challenges (*n* = 12)

Participants identified barriers related to mental health service delivery systems, including financial and insurance-related challenges, provider shortage and turnover, system failures, and provider characteristics. One participant identified language as a potential barrier.

Insurance-related challenges and an inability to pay for services were cited as barriers in all focus groups. One described the primary barrier to treatment as “Economics, first for some… they do not help themselves because they do not have the financial means to do so.” Another participant stated:

Many do not have medical insurance or some help they do not accept; they cannot get a doctor for treatment because they do not have medical insurance or they cannot pay even half or according to their resources they would have to pay this and if they cannot pay even that, they can’t help you.

One participant shared, “… those that do ask for insurance, if they do not have insurance, they do not take it now, anywhere, if you do not have insurance, you do not get it.” Another identified as a barrier, “Money… Money and lack for those who have insurance there are no psychiatrists… they do not get this insurance or they do not get the other one and it’s impossible.” In reflecting upon why someone would not seek services, one participant shared:

In our Latino community, the big barrier right now is money… That is very expensive, one has the mentality that it is going to be very expensive and yes, it’s expensive, but that’s one of the reasons, paying for the treatment.

Another described a friend who “came to therapy and got his whole family involved and started spending a lot of money, got excited… then the time came when he said ‘I cannot anymore, it’s too much money.’”

Provider shortage, turnover, and waiting times were frequent points of discussion. One participant shared, “There aren’t that many therapists, there aren’t that many people…” Another shared:

There are not enough doctors for the people who need help and the doctors that there are… are overbooked or they do not take this insurance, we do not take the other one, they only send it to [clinic name omitted] … if you go over there, they lock you up there.

In reflecting upon turnover, one participant shared, “It’s very difficult mentally because you have already opened up with one person, they leave in 2 years, and then you have to start over and then with another.” One participant in the same focus group responded, “And then with another person it is to explain everything again, right? That’s how it is at [clinic name omitted], they put you with one person for 6 or 8 months and then they change him with another.” Another described it as:

Or they have a lot of people, they say… I didn’t get the insurance anymore, okay, they give me a referral to [clinic name omitted] for 2 years to go to this place to see a psychiatrist… the problem of when there are people with fewer resources who send us to places like this… it is very difficult to find help because not everyone can help us.

Focus group participants shared additional observations about broken referral systems and processes. One participant described, “They gave me three numbers here and then I tried and they did not answer me at all, if not, they came back about 2 months later… it’s that they have not answered me in these numbers.” Another stated,

They gave them a number here for different doctors, and she calls and nobody answers, so why give her number if no one is going to answer. I could give you 100 numbers and nobody answers so what is the purpose?

A handful of participants mentioned provider characteristics as barriers. One participant described discrimination as a potential barrier with providers paying “attention more in the Americans, in the white male,” causing a person to withdraw from treatment. Someone rebutted:

I think it’s not because of color, or how to say a religion or nationality, a doctor doesn’t see that, he sees the patient and what the patient is suffering and I personally have been treated the same. I consider myself Mexican, but all the time they have treated me the same as other people.

It was clear that participants were also considering goodness of fit between themselves and mental health providers. One participant described a therapist not meeting her where she was at, “I do not understand why they have to corner you when I go to therapy, it’s because I want to, so I go and drop everything, everything I bring to receive help, but that person saw it [a different] way.” Another stated,

I felt like pressure and she wanted me to. I felt like she was pushing me a little too much… she is not seeing, or she is not listening where am I at that she is pushing me to get the goal… I felt pressure.

Whether systemic, individual-level, or stigma-related, the barriers participants shared provide insight into the challenges Hispanic adults in the U.S.-Mexico border region experience when seeking and accessing mental health services.

### 3.3. Theme: mental health treatment facilitators (*n* = 7)

Some participants identified factors that promote help-seeking and retention in mental health services. These primarily encompassed motivation and utilization of natural supports. Other treatment facilitators can be implied through previous sections on understanding mental health and barriers to seeking and receiving services.

#### 3.3.1. Subtheme: motivation (*n* = 3)

Three participants described motivation as a facilitator of mental health treatment utilization and retention, reflecting the importance of intrinsic motivation and self-determination. One participant shared, “The person who wants to regenerate, regenerates… the consistency of the person who wants to improve himself, who wants to get out of what he brings, of his problems.” Another stated that people are more likely to stay in treatment if they “see improvement, [have an] attitude change.” Lastly, a participant described “motivation towards something that works, to continue using it… use what works for you, if you already know what works for you, use it.”

#### 3.3.2. Subtheme: natural supports (*n* = 4)

The importance of family support was similarly cited as a facilitator of treatment. One participant described “family support, more than anything” as critical to supporting someone’s mental health recovery. Another participant shared,

The support of the family is very helpful, because they see that the family is with them and the more the family is united, the faster they come out of what they go through because they see the family unit there is. On the other hand, if they leave them, then I won’t go with you or I’ll take you later. It helps a lot that the family is supporting them.

One participant shared, “The family too… if they are in treatment, and they think of their family, that is why they continue with treatment, to return to their family, to recover their family.” In addition to family, another participant shared, “I go to support groups… there are a lot of support groups all around.”

The mental health facilitators described by participants reflect intrinsic and extrinsic sources of motivation, and the cultural importance of family.

### 3.4. Theme: recommendations for agencies, providers, and researchers (*n* = 16)

Participants made several recommendations to promote help-seeking and improve access to mental health services. These included increasing outreach, providing more direct education, engaging the community in awareness building and research, and improving aspects of mental health service delivery systems.

#### 3.4.1. Subtheme: outreach and education (*n* = 5)

Many participants stressed the importance of public awareness and educational campaigns to increase community knowledge of mental health and available resources. One participant suggested that providers “make more brochures, put up brochures, and go out into the community to make them understand that there are places… [that] can help them.” Another participant agreed, “it is good that they put up little signs like that… do you need help? You can talk to this person.” This participant continued:

Let’s go out like as a community that we are interested in the other neighbors so we are going to give a little pamphlet… it would be very interesting for people to see information about this wherever they go, so that they have knowledge that there are agencies that can help them.

Others similarly emphasized the importance of spreading the word about mental health conditions and potential treatment options. One stated:

When you go to the hospitals or enter the bathrooms and they tell you, “do you feel safe at home?” And if you don’t, you get an 800 number and call, right? Then why not put, “you feel drowned with your thoughts?” … you can call someone too.

Another participant shared about attending educational classes in Mexico for physical health conditions where “they also put a class, they did not say it at first. They said it was more for… those three [physical] problems… but they also included a psychologist and then they created a space without openly saying it.”

One participant recommended more explicitly that mental health providers offer more education about mental health conditions, sharing, “I’d like to be more educated as far as what it is that I suffer from or what is it that I can do…” He continued:

It needs to start earlier so that way obviously kids know [what] the symptoms are… show what exactly symptoms of the common anxiety, depression, PTSD… the ones a lot of people are dealing with. I would say educate them as far as what are these symptoms, what is it that you are feeling, so people can recognize it.

#### 3.4.2. Subtheme: improving mental health services (*n* = 13)

While participants emphasized providing more education, several participants also suggested specific improvements to mental health service delivery systems, including increasing resources and funding, integrating mental health services in other settings, providing more client-driven care, utilizing existing resources, and creating new treatment opportunities.

Given that financial and insurance-related challenges were prominent barriers identified by focus group members, it is understandable that participants had recommendations related to overcoming this challenge. One participant recommended ensuring “that insurance is not a problem to reach that [mental health] resource.” Another identified a need for “more resources for low-income people, those who do not have insurance and do not have money…. And the government should provide money for these things that are very important… instead of going to build a better wall.” This participant similarly noted the importance of increasing access through reducing extensive waitlist times. Another participant similarly recommended “being flexible in the schedule… their appointments are very full, then it is very difficult [to access services].” One participant suggested “free counseling services to those in need.” A participant from a separate focus group similarly suggested, “Since this is something that concerns the entire country, I think it would be a good idea for the country to invest funds to make the visit to the therapist more accessible.”

One participant recognized this as a need for more agencies, “more rehabilitation centers have to be opened so that all people with needs have access.” Another participant shared, “… we need more of these community services so that in case they cannot afford one, or the insurance does not catch you here, then there should be extra help.” One participant described this as, “help without so many requirements, because sometimes they ask for so many requirements that for this reason, many people cannot.”

Other participants provided additional recommendations for improving mental health service delivery systems, with a handful touching on integrating mental health services in other settings. One participant shared, “My advice would be that those people who want to start doing this help… that they be integrated right now in clinics that are for the community.” Another participant shared:

Take advantage of the possible groups that already exist with other reasons that have to do not with mental health, that have to do with perhaps physical health. I think that already there are those groups and… focus on those groups that already exist and add that part to them.

Across focus groups, participants discussed rethinking how we engage people in mental health services. One participant recognized that individuals with mental health issues are “not going to approach you [providers],” making a case for more active engagement and outreach. This participant shared the importance of personally inviting people into services, “we can invite someone else and here it says here, we have this if you have any problem, you can call and it’s something important.” Another participant shared:

…an agency or social worker… when they know of a person who has problems… call them or visit their home to find out how they are, or a simple greeting… are you okay, or what do you think if I give a little visit to see how you are doing? How does he feel?

Meeting clients where they are at, and treating them as individual human beings, was a common theme. One participant suggested, “not [seeing people] with dollar signs.” Another responded, “that they see people as people, not as a patient… they have the heart of compassion for people.” One participant reflected on negative interactions with providers, “many times when something is already negative, and you listen to something you do not like, you say it’s over. But as long as it’s being positive, that’s what’s going to continue.” One participant shared frustration with providers prioritizing medication as the treatment option:

…they just want to give you medication… how about I try something else before we resort to that, why don’t you educate me about it, and figure out what we can do, so that way I learn other things to do.

One participant shared the importance of obtaining community endorsement for services, “look for people who were leaders in communities and neighborhoods” to bring more people into mental health services.

#### 3.4.3. Subtheme: community engagement and research (*n* = 7)

Participants shared openly about their experiences participating in studies like this and the importance of engaging communities in research to improve the community and individual access to mental health services. One participant shared, “The community really needs help, and thanks to you for these studies that you are doing… because we can focus on something that is going to change, that is going to come.” Another participant observed through participating in this study, “that the people be concerned, what they are doing is very good, that they are worrying about us for everything, for the community, that is very good.” Another shared:

You don’t just come to come, you come because you want to learn from us. And we don’t know anything about you … we are going to need people like you to come here to open our eyes to continue moving forward, better than worse.

Another participant suggested involving others in this line of research:

… look for information on whether there is someone else interested in the same topic so that it is… stronger on the subject… it would be better because the system was more complete and it would be of more help both for the person who is going to do it and for those who are going to receive the help.

Others similarly suggested expanding the scope of research and fostering dialogue between communities and researchers. One suggested that researchers “should try to do this, but with the same people who need the help, to see what their opinion is too.” Another suggested:

Why not do it with a wider community? If you speak with more groups, you share [with us] and so we know what is happening… if you do not share anything with us, then you will only know the theory of us.

## 4. Discussion

Research explicitly focusing on the unmet mental health needs and mental health experiences of Hispanic populations is limited. The present study explored the unique and personal experiences of Hispanic adults, primarily of Mexican descent, living in the Paso del Norte U.S.-Mexico border region. A significant finding in this study is the relationship between cultural beliefs and values and mental health stigma as a barrier to talking about mental health and seeking mental health services. For some participants, seeking mental health treatment was seen as a weakness. These findings are consistent with previous literature on factors that broadly impact help-seeking behaviors in Hispanic populations (12, 25).

Participants shared specific nuances around how their perspectives and viewpoints evolved though living in a binational bicultural community. Many of our participants have lived experiences on both sides of the border, a unique contribution of this study. For some, the experience of crossing the U.S.-Mexico border provided exposure to different ways of thinking about mental health that then impacted their own thinking. Several participants shared their perspectives of mental health becoming less stigmatizing on the U.S. side of the border, carrying potential implications for an association between length of time in the U.S. and mental health stigma.

Our findings also align with previous research on general mental health treatment experiences and utilization for Hispanic populations. Language barriers, unfamiliarity with cultural beliefs, and an inability to obtain insurance or afford treatment were all cited as barriers for our study population, consistent with previous findings on mental health treatment utilization for Hispanic populations (26). Other barriers included a lack of time, competing priorities, and a lack of awareness and education. Throughout our conversations on barriers, prominent underlying factors included poverty, stigma, and shame. These frequently stem from systemic racism, sexism, and colonization (27), often resulting in collective oppression-based traumas that disproportionately impact communities of color (28) and shape how they view and seek professionalized services.

Participants expressed a wide range of views of mental health service systems and treatment providers, often reflecting a distrust of the system. The present thematic findings suggest mostly negative perceptions related to (1) feeling judged or pressured by providers, (2) being routed to restrictive services like involuntary commitments, (3) feeling that systems are focused more on money than helping, (4) working with providers who are quick to jump to medication when less restrictive options are preferred, (5) service delivery systems that are difficult to navigate, (6) long wait times to be seen, and (7) frequent provider turnover. Addressing these systemic barriers will be critical as we seek to improve service delivery systems for all populations.

Participants also identified factors that help people seek and stay in treatment. Experiencing a perceived positive benefit of treatment was cited by multiple participants in promoting treatment adherence and success. Natural supports, including the encouragement and assistance of friends, family, and support groups were also seen as critical to finding the motivation to seek treatment and being successful in treatment, consistent with existing literature (29). Participants shared many recommendations for encouraging help-seeking and improving mental health services, which are highlighted below.

### 4.1. Practice and policy recommendations

In addition to learning about participants’ understanding and experiences with mental health and treatment, our next step is to use these findings to develop an innovative, culturally responsive mental health engagement program. Recognizing that evidence-based practices are often inadequate in meeting the needs of communities of color, we have adopted a “bottom-up” approach to program development and implementation (30). We know that no culture is homogenous, and treatment providers and program developers should engage potential recipients of services, as we did in this study, to better understand how program components may or may not align with cultural beliefs, values, and practices.

The cultural responsiveness of any intervention is critical to its success. Barrio (31) recommends that eliciting family support and providing group modalities may be most effective for cultures reflective of socio-centric thinking. In our study, this concept was evident in the sense of community belonging developed amongst focus group members, in the identification of family support as so critical to treatment success, and in the value gained through helping those in their lives with mental health challenges. A collectivist orientation was prominent throughout the conversations on help-seeking and should be emphasized in treatment approaches.

In honoring historical and present cultural trauma, program development and implementation should recognize the impacts of racism, sexism, and colonization on Hispanic adults’ perceptions of professional help systems. This approach will better equip the mental health treatment community to incorporate culturally responsive practices into their treatment. Some practical strategies for achieving this include fostering interpersonal relationships, promoting a collaborative model of care, providing education on mental health and stigma, utilizing natural supports, including religion and spirituality, and ensuring culturally responsive providers (29). These strategies are all consistent with recommendations made by our focus group participants.

Above ensuring the cultural responsiveness of mental health programs, systemic barriers need to be addressed through policy and practice. While not unique to any one population, communities of color and those with lower socioeconomic statuses tend to be disproportionately affected by these barriers, as evidenced by less frequent help-seeking and treatment utilization (1). Focus group participants recommended the following strategies to improve mental health service delivery systems: addressing financial and insurance barriers, providing more client-centered care, growing mental health treatment resources, providing mental health care integrated within other settings, and providing more community engagement and outreach. These are all tangible improvements that can be made if supported by funding, policy, and practice guidelines.

### 4.2. Strengths and limitations

The methodological approaches taken in this study add to its overall strengths in three major ways. First, the involvement of a community agency in study development and research design increased the likelihood of methods and content being responsive to community needs. Second, help-seeking behaviors and treatment engagement often exclude Spanish-speaking participants altogether (13). All research was conducted in Spanish, English, or a mix of English and Spanish to honor participants’ preferred language. Last, the research team shared similar ethnic characteristics and spoken language to the study population. Graduate research assistants who collected data were fully bilingual and from the same region as the participants. This approach helped foster trust and connection between researchers and participants throughout the study.

Despite these strengths, our study findings have limitations. Since all participants were recruited from a local food pantry, their perspectives may reflect only some of the larger community’s perspectives on mental health and barriers to seeking and accessing care. Participants were also asked to share their views and predict the views of families, friends, and other community members. As such, some of the qualitative content may not reflect the participants’ personal beliefs. Nevertheless, by requesting multiple perspectives and points of view, researchers collected rich data to reflect patterns and trends beyond just the focus group member experiences.

## 5. Conclusion

In this study, we strived to engage community providers and community members as active participants in this research, promoting a co-learning process and facilitating dialogue around the role that research can play in improving individual and community wellness (32). The rich conversations from these focus groups can be attributed mainly to the cultural congruence of focus group facilitators, meeting participants in a community-based agency, and providing the groups in participants’ preferred language. Participants regularly shared gratitude for the research being conducted, sharing so openly about challenges they have experienced and providing strategies they believe will improve mental health help-seeking and treatment access for Hispanic adults in the Paso del Norte U.S.-Mexico border region. While there are several barriers to seeking help and accessing treatment, there is also a strong commitment to engaging in the work necessary to reduce stigma, improve access, and eliminate the mental health treatment disparities experienced by Hispanic populations.

## Data availability statement

The raw data supporting the conclusions of this article will be made available by the authors, without undue reservation.

## Ethics statement

The studies involving human participants were reviewed and approved by the Institutional Review Board at the University of Texas at El Paso. The patients/participants provided their written informed consent to participate in this study.

## Author contributions

JM, RE, and CT contributed to the conception and design of the study. RE was the primary facilitator of the focus groups and translated and transcribed focus group content. JM organized all data and wrote the first draft of the manuscript. JM and RE performed initial independent data analysis. ER and CT cross-checked raw data with findings and provided recommendations for strengthening validity of coding framework. ER provided cultural content expertise during data analysis and manuscript preparation. CT provided community practice content expertise during data analysis and manuscript preparation. RE, ER, and CT contributed unique content to various sections of the manuscript. All authors contributed to manuscript revision, read, and approved the submitted version.

## Funding

This work was partially funded through a University Research Institute grant from The University of Texas at El Paso and a Sobel-Duncan Health Disparities Research Award.

## Conflict of interest

The authors declare that the research was conducted in the absence of any commercial or financial relationships that could be construed as a potential conflict of interest.

## Publisher’s note

All claims expressed in this article are solely those of the authors and do not necessarily represent those of their affiliated organizations, or those of the publisher, the editors and the reviewers. Any product that may be evaluated in this article, or claim that may be made by its manufacturer, is not guaranteed or endorsed by the publisher.

## Footnotes

^1^The term Hispanic as an ethnonym was chosen by the research team to encompass anyone of Latin American descent who may be living in the border region and participating in this research. The research team recognizes the limitation of using this as an umbrella term and therefore asked participants to provide further information about their ethnicity in an open-ended question.
